# The Metabolite Profile in Culture Supernatant of *Aster yomena* Callus and Its Anti-Photoaging Effect in Skin Cells Exposed to UVB

**DOI:** 10.3390/plants10040659

**Published:** 2021-03-30

**Authors:** Woo Sik Kim, Jeong Hun Seo, Jae-In Lee, Eun-Sil Ko, Sang-Min Cho, Jea-Ran Kang, Jong-Hoon Jeong, Yu Jeong Jeong, Cha Young Kim, Jeong-Dan Cha, Young-Bae Ryu

**Affiliations:** 1 Functional Biomaterial Research Center, Korea Research Institute of Bioscience and Biotechnology, Jeongeup-si 56212, Korea; kws6144@kribb.re.kr (W.S.K.); lji613@kribb.re.kr (J.-I.L.); yjjeong@kribb.re.kr (Y.J.J.); kimcy@kribb.re.kr (C.Y.K.); 2 Department of Bio-Material and Product Development and R&D Center, General Bio, Namwon-si 55793, Korea; ceo@generalbio.co.kr (J.H.S.); kes@generalbio.co.kr (E.-S.K.); csm@generalbio.co.kr (S.-M.C.); kjr@generalbio.co.kr (J.-R.K.); jhjeong@generalbio.co.kr (J.-H.J.)

**Keywords:** *Aster yomena* callus, metabolite profile, photoaging, UV exposure, skin diseases

## Abstract

*Aster yomena* (*A. yomena*) extract has anti-inflammatory, antioxidant, anti-asthma, and anti-atopic effects. However, the commercial use of *A. yomena* extract requires a long processing time with specific processing steps (including heat treatment and ethanol precipitation), and there are various environmental problems. We aimed to build a system to produce *A. yomena* extract by culturing the callus in a bioreactor that can allow rapid process scale-up to test the effect of extract (AYC-CS-E) isolated from culture supernatant of *A. yomena* callus on photoaging of human keratinocytes (HaCaT) caused by ultraviolet B (UVB) exposure. Through screening analysis based on ultra-performance liquid chromatography-quadrupole time-of-flight mass spectrometry (UPLC/Q-TOF-MS), 17 major metabolites were tentatively identified from AYC-CS-E for the first time. The suppression of cell proliferation caused by UVB was effectively alleviated in UVB-irradiated HaCaT cells treated with AYC-CS-E. Treatment with AYC-CS-E strongly induced the formation of type I procollagen and the inhibition of elastase in UVB-irradiated HaCaT cells and significantly reduced the expression of matrix metalloproteinase (MMP)-1. In addition, treatment of UVB-irradiated HaCaT cells with AYC-CS-E effectively improved various factors associated with an inflammatory reaction, skin damage recovery, skin moisture retention, and hyper-keratinization caused by photoaging, such as reactive oxygen species (ROS), pro-inflammatory cytokines, transforming growth factor beta (TGF-β), MMP-3, MMP-9, filaggrin, hyaluronic acid synthase 2 (HAS-2), keratin 1 (KRT-1), nuclear factor-kappa B (NF-κB), and nuclear factor erythroid 2-related factor 2 (Nrf2) at the gene and protein levels. These results suggest that AYC-CS-E can be used as a cosmetic ingredient for various skin diseases caused by photoaging, and the current callus culture system can be used commercially to supply cosmetic ingredients.

## 1. Introduction

Natural resources have been widely employed as cosmetic ingredients for many years [1,2]. Natural resources are used in traditional medicine and as raw materials for cosmetics, which is considered to be an effective marketing strategy [3,4]. Numerous studies have reported the positive effects of natural resources on human skin, such as antioxidant, anti-aging, anti-inflammatory, whitening, and anti-wrinkle effects [5,6,7]. Despite these benefits, natural resources face extinction and pollution [8]. For instance, global climate change due to deforestation and consumption of fossil fuels has a direct impact on natural resources [9]. In addition, inhibition of growth and mutation may be induced by toxic chemicals, air pollution, and water pollution [10,11]. These phenomena can have negative effects in various industrial fields that require a large number of natural resources of uniform quality. Therefore, there is an urgent need for a technology to harvest a large amount of high-quality natural resources, regardless of the growing environment, such as the region, temperature, and cultivation conditions.

Plant tissue culture has been used for the induction of somatic cells, mass proliferation, and development of transgenic plants, and plant cell culture has been reported as a future-oriented technology that can improve the productivity of effective substances in plants [12,13,14]. In particular, a plant callus culture system has the advantage of stable and rapid plant cell generation under constant culture conditions [14,15]. Plant callus, also called a plant stem cell, is a tissue that divides vigorously around the wound when the plant body is damaged [15,16,17]. Callus, actively engaged in cell division, contains a high concentration of physiologically active substances with anti-inflammatory, antioxidant, anti-wrinkle, and whitening effects [15,18,19]. As plant callus cells contain a variety of physiologically active substances that are effective for human skin, unlike plant bodies that are completely differentiated, they can be used as a core raw material for cosmeceuticals and biocosmetics [15,16,18]. In addition, this system is being considered as an efficient approach for producing natural compounds for pharmaceutical applications [20]. Interestingly, the Samyang Genex (Taejon, Korea) has established a callus culture system that can consistently produce paclitaxel, a known anti-cancer drug, and it (commercial name is Cynviloq^TM^) is currently used as a commercial drug for ovarian, breast, and pancreatic cancers [20,21]. Therefore, callus culture system could be usefully applied not only to the cosmetic industry but also to various pharmaceutical industries.

*Aster yomena* (*A. yomena*) is an edible vegetable and a perennial herb that is found in many regions, such as south Korea, China, Japan, and Siberia, and has been used as a raw material for folk remedies to cure cough, bronchial asthma, and insect bites [22]. Recently, the Korea Food and Drug Administration registered a leaf extract of *A. yomena* as a functional food in the Korean Food Standards Codex, which is being sold as a functional food for the prevention or alleviation of allergy symptoms. This registration was based on research findings (anti-allergic effects of *A. yomena* extract using a mouse allergy model and cells) from Hwang et al. [23]. In addition, various researchers have demonstrated the anti-inflammatory, antioxidative, and anti-asthma functions of the *A. yomena* extract [22,24]. In this respect, we predict that the callus culture system of *A. yomena* can have a high market value in the development of cosmetics and functional foods. Moreover, inducing *A. yomena* callus will allow harvesting a large number of materials with high purity, high efficiency, and high functionality while resolving the environmental issues associated with the process.

Ultraviolet (UV) light produced by the sun can induce extensive skin damage because it has enough energy to cause photochemical injury to cellular systems [25]. Among the types of UV radiation, ultraviolet B (UVB) rays can induce damage to the cellular DNA and are the primary cause of skin photoaging [26,27]. In the worst cases, they can cause skin diseases such as actinic keratosis and skin cancer [28]. In this study, extract (AYC-CS-E) isolated from culture supernatant of *A. yomena* callus obtained by using a bioreactor system for industrial scale-up of a callus production system was used to examine its effect on the inhibition of photoaging of human keratinocytes (HaCaT) caused by UVB exposure, aiming to confirm its effectiveness as a new natural functional cosmetic ingredient. 

## 2. Results

### 2.1. Identification of Major Metabolites from AYC-CS-E

**Figure 1:**
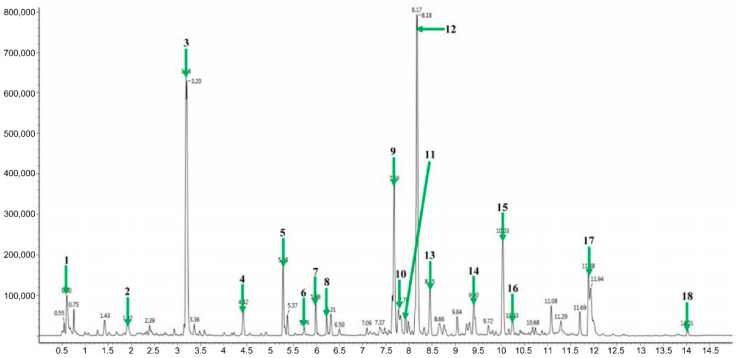
Representative ultra-performance liquid chromatography-quadrupole-time-of-flight mass spectrometry (UPLC-QTOF/MS) of extract (AYC-CS-E) isolated from culture supernatant of *Aster yomena* callus. For analysis, the metabolites were analyzed with a BEH C18 column (2.1 × 100 mm, 1.7 μm). The eluted metabolites were analyzed by Q-TOF/MS in electrospray (ESI)-positive mode. The U PLC-QTOF/MS chromatogram shows the following: Green arrow 1, Robustic acid; green arrow 2, 3,4-Dicaffeoyl-1,5-quinolactone; green arrow 3, Acetylpterosin C; green arrow 4, Pterosin N; green arrow 5, L-thyronine; green arrow 6, 3,5-Di-O-methyl-8-prenylafzelechin-4beta-ol; green arrow 7, Dehydrophytosphingosine; green arrow 8, Phytosphingsoine; green arrow 9, LysoPC(18:2); green arrow 10, α-Linolenic acid; green arrows 11 and 12, LysoPC(16:0); 13, LysoPC(18:1); green arrow 14, LysoPC(18:0); green arrow 15, Palmitic amide; green arrow 16, Oleamide; green arrow 17, 13Z-Docosenamide; green arrow 18, PC(18:2/16:0).

Many studies have reported that plants can produce various secondary metabolites as natural antioxidants (including flavonoids and isoflavones, etc.) to protect the skin from photoaging [29,30]. Thus, we identified metabolites that may be correlated with their pharmacological activities in AYC-CS-E derived from *A. yomena* roots. In fact, the formation of these calluses has also been attempted on leaves and stems, but the callus was most effectively formed in the roots (data not shown). The extraction method of functional materials from callus pellets requires several processing steps, such as heat treatment and methanol or ethanol precipitation [31]. Here, the heat treatment step can cause denaturation and loss of functional materials [32]. Thus, AYC-CS-E, which can be prepared by minimizing material damage and processing time, isolated from callus culture system using *A. yomena* roots was used in this study. Metabolites of AYC-CS-E were analyzed by liquid chromatography/mass spectrometry (LC/MS; Figure 1). As a result, 17 major metabolites—including 1 isoflavonoid (robustic acid), 1 flavonoid (3,5-Di-O-methyl-8-prenylafzelechin-4beta-ol), 2 indanes (acetylpterosin C and pterosin N), 1 amino acid (L-thyronine), 1 cinnamic acid derivative (3,4-dicaffeoyl-1,5-quinolactone), 2 sphingolipids (dehydrophytosphingosine and phytosphingosine), 4 fatty amides (α-linolenic acid, palmitic amide, olemaide, and 13Z-docosenamide), and 5 glycerophospholipids (LysoPC(18:2), LysoPC(16:0), LysoPC(18:1), LysoPC(18:0), and PC(18:2/16:0)) were identified from AYC-CS-E for the first time (Table 1). 

### 2.2. Cytoprotective Effect of AYC-CS-E on UVB-Irradiated HaCaT Cells

**Figure 2:**
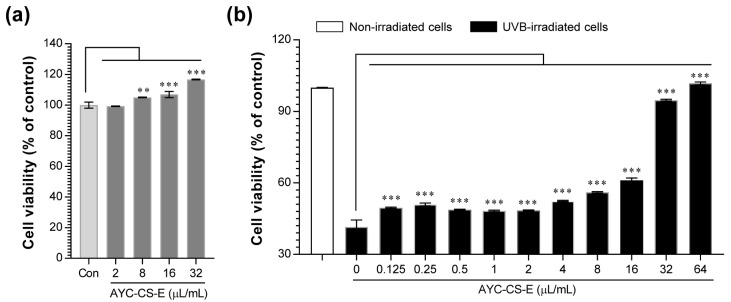
Effect of AYC-CS-E treatment on cell viability of non- and ultraviolet B (UVB)-irradiated human keratinocytes (HaCaT cells). (**a**) The HaCaT cell line at a concentration of 1 × 10^5^ cells/well was seeded in a 48-well plate, incubated for 12 h in an incubator at 37 °C and 5% CO_2_, and treated with the indicated concentrations (2, 8, 16, or 32 μL/mL) of AYC-CS-E (30 mg/mL) for 24 h. The cell viability was measured using 3-(4, 5-dimethylthiazol-2-yl)-2, 5-diphenyl-tetrazolium bromide (MTT) assay. Con: non-treated HaCaT cells, (**b**) The cells (1 × 10^5^ cells/well in 48-well plate) were treated with various concentrations (0.125 to 64 μL/mL) of AYC-CS-E for 24 h after being exposed to 8 mJ/cm^2^ UVB irradiation, as described in the Materials and Methods section. The cell viability for each condition was measured using the MTT assay. All bar graphs show the means ± standard deviation (SD) of 3 samples. One representative plot out of three independent experiments is shown. ** *p* < 0.01, and *** *p* < 0.001.

UVB exposure of the skin promotes DNA damage in skin cells and the induction of apoptotic cell death with irreparable DNA damage caused by UVB [33]. To investigate the protective effect of AYC-CS-E on UVB-irradiated HaCaT cells, we first evaluated the cell viability of HaCaT cells after treatment with AYC-CS-E alone. Then, its cytoprotective effect was further assessed on UVB-irradiated HaCaT cells. AYC-CS-E increased the proliferation of HaCaT cells in a concentration-dependent manner (Figure 2a). The cytoprotective effect of AYC-CS-E on UVB-irradiated HaCaT cells was examined based on the effect of AYC-CS-E alone on cell viability, revealing that cell proliferation was further increased in all UVB-irradiated HaCaT cells treated with 0.125 to 64 µL/mL AYC-CS-E (Figure 2b). These results suggested that AYC-CS-E alleviated the cell damage caused by UVB.

### 2.3. Elastase Inhibition, Type I Procollagen Synthesis Promotion, and MMP-1 Inhibition Effects of AYC-CS-E

**Figure 3:**
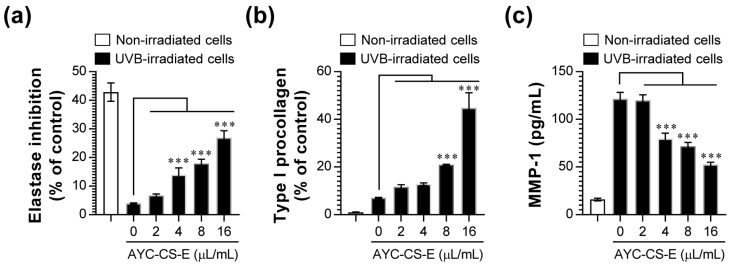
Effect of AYC-CS-E treatment on elastase inhibition, type I procollagen expression, and matrix metalloproteinase-1 (MMP-1) production of UVB-irradiated HaCaT cells. (**a–c**) HaCaT cells were treated with various concentrations (2, 4, 8, or 16 μL/mL) of AYC-CS-E (30 mg/mL) for 24 h after being exposed to 8 mJ/cm^2^ UVB irradiation. (**a**) Elastase inhibition levels were measured in each condition via elastase substrate (N-STANA; N-succinyl-tri-alanyl-p-nitroanilide) treatment, as described in the Materials and Methods section. Type I procollagen (**b**) and MMP-1 (**c**) levels in the culture supernatants were analyzed using enzyme-linked immunosorbent assay (ELISA) kits. All bar graphs show the means ± standard deviation (SD) of 3 samples. One representative plot out of three independent experiments is shown. *** *p* < 0.001.

UVB not only hinders type I procollagen synthesis via an increase in matrix metalloproteinase-1 (MMP-1) in the human skin, but also promotes the degradation of elastin [34]. These phenomena can lead to skin photoaging. Based on these results, we investigated the effect of AYC-CS-E treatment on photoaging by examining elastase inhibition, promotion of type I procollagen synthesis, and MMP-1 inhibition upon AYC-CS-E treatment in UVB-irradiated HaCaT cells. As a result, elastase inhibition (Figure 3a), promotion of type I procollagen synthesis (Figure 3b), and MMP-1 inhibition (Figure 3c) were induced in a concentration-dependent manner in UVB-irradiated HaCaT cells treated with AYC-CS-E.

### 2.4. Expression of Photoaging Regulators Induced by AYC-CS-E

**Figure 4:**
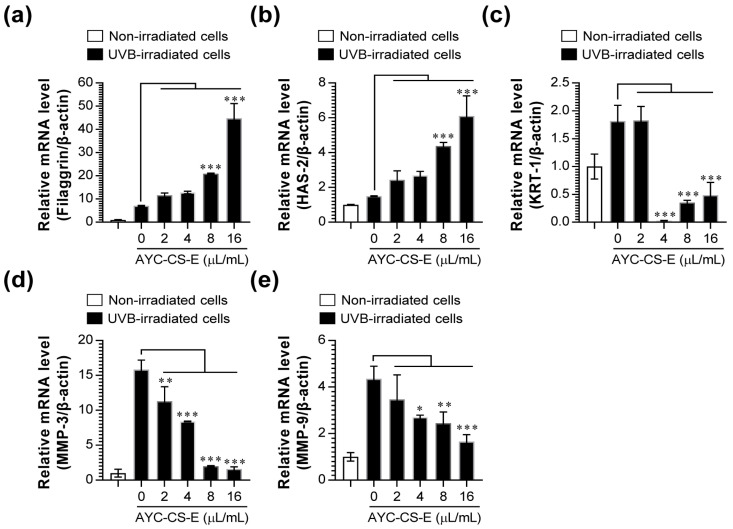
Effect of AYC-CS-E treatment on photoaging-related factor expression of UVB-irradiated HaCaT cells. (**a**–**e**) HaCaT cells were treated with various concentrations (2, 4, 8, or 16 μL/mL) of AYC-CS-E (30 mg/mL) for 24 h after being exposed to 8 mJ/cm^2^ UVB irradiation. Cells were collected, total RNA was extracted, and cDNA was synthesized for quantitative real-time polymerase chain reaction (qRT-PCR), as described in the Materials and Methods section. The mRNA expression of the photoaging-related factors (Filaggrin (**a**), HAS-2 (**b**), KRT-1 (**c**), MMP-3 (**d**), and MMP-9 (**e**)) were analyzed by a real-time PCR machine. HAS-2: hyaluronic acid synthase 2; KRT-1: keratin 1. Relative mRNA expression levels were normalized to β-actin. All bar graphs show the means ± standard deviation (SD) of 3 samples. One representative plot out of three independent experiments is shown. * *p* < 0.05, ** *p* < 0.01, or *** *p* < 0.001.

Next, changes in filaggrin, hyaluronic acid synthase 2 (HAS-2), keratin 1 (KRT-1), MMP-3, and MMP-9 expression, which are important factors associated with photoaging [35,36,37], were analyzed at the gene level. After examining the mRNA expression of filaggrin, HAS-2, KRT-1, MMP-3, and MMP-9 in UVB-irradiated HaCaT cells treated with AYC-CS-E, we found that the mRNA expressions of filaggrin (Figure 4a) and HAS-2 (Figure 4b) were higher in UVB-irradiated HaCaT cells treated with AYC-CS-E than those in UVB-irradiated HaCaT cells not treated with AYC-CS-E. Moreover, the increase in the mRNA expression of KRT-1 (Figure 4c), MMP-3 (Figure 4d), and MMP-9 (Figure 4e) in UVB-irradiated HaCaT cells was suppressed upon treatment with AYC-CS-E.

### 2.5. Anti-Inflammatory and Antioxidant Effect of AYC-CS-E Treatment on UVB-Irradiated HaCaT Cells

**Figure 5:**
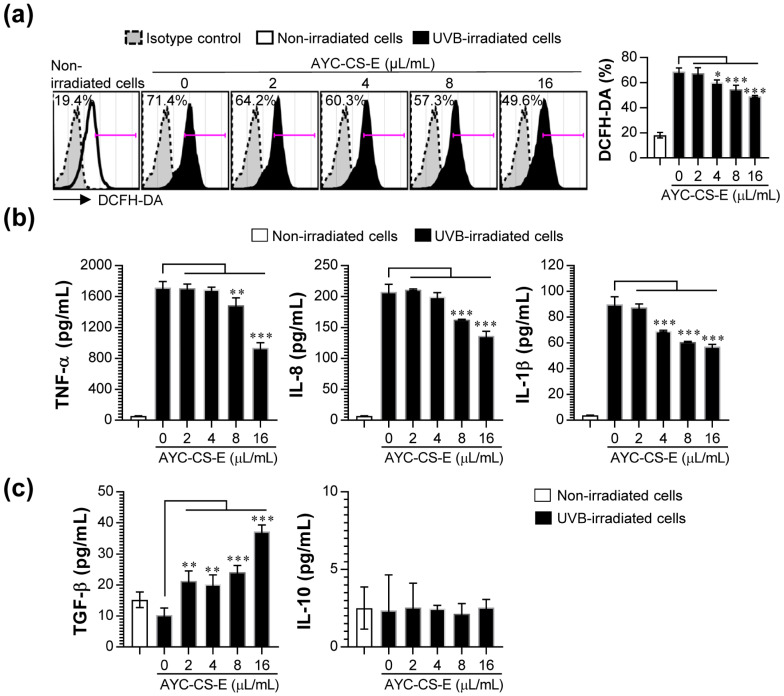
Effect of AYC-CS-E treatment on reactive oxygen species (ROS) production, and pro- and anti-inflammatory cytokine secretion of UVB-irradiated HaCaT cells. (**a**–**c**) HaCaT cells were treated with various concentrations (2, 4, 8, or 16 μL/mL) of AYC-CS-E (30 mg/mL) for 24 h after being exposed to 8 mJ/cm^2^ UVB irradiation. (**a**) Cells were harvested, and intracellular ROS levels were analyzed by 5 μM 2′,7′-dichlorofluorescein diacetate (DCFH-DA) staining, as described in the Materials and Methods section. (**b**,**c**) The culture supernatants were collected, and pro- ((**b**) Tumor necrosis factor-α (TNF-α), Interleukin-8 (IL-8), and IL-1β) and anti-inflammatory cytokine ((**c**) transforming growth factor beta (TGF-β) and IL-10) levels were analyzed using cytokine-specific ELISA kits. All bar graphs show the means ± standard deviation (SD) of 3 samples. One representative plot out of three independent experiments is shown. * *p* < 0.05, ** *p* < 0.01, or *** *p* < 0.001.

The exposure to UVB irradiation in the skin may result in excessive production of reactive oxygen species (ROS) and acute inflammatory responses from keratinocytes, thereby resulting in the induction of erythema and cell apoptosis [38,39]. Interestingly, these symptoms can be improved by adding anti-oxidative and/or anti-inflammatory compounds [40]. Therefore, we evaluated whether the production of excessive ROS and various pro-inflammatory cytokines (tumor necrosis factor-α (TNF-α), Interleukin-8 (IL-8), IL-1 beta (IL-β)) in UVB-irradiated HaCaT cells are inhibited upon treatment with AYC-CS-E. Treatment with AYC-CS-E effectively inhibited the production of intracellular ROS (Figure 5a) and pro-inflammatory cytokines (TNF-α, IL-8, and IL-1β; Figure 5b) in UVB-irradiated HaCaT cells. In addition, UVB-irradiated HaCaT cells treated with AYC-CS-E increased the secretion of transforming growth factor beta (TGF-β, which is an anti-inflammatory cytokine), which can improve oxidative stress and pro-inflammatory responses compared to UVB-irradiated HaCaT cells not treated with AYC-CS-E, but not IL-10 production (Figure 5c). 

**Figure 6:**
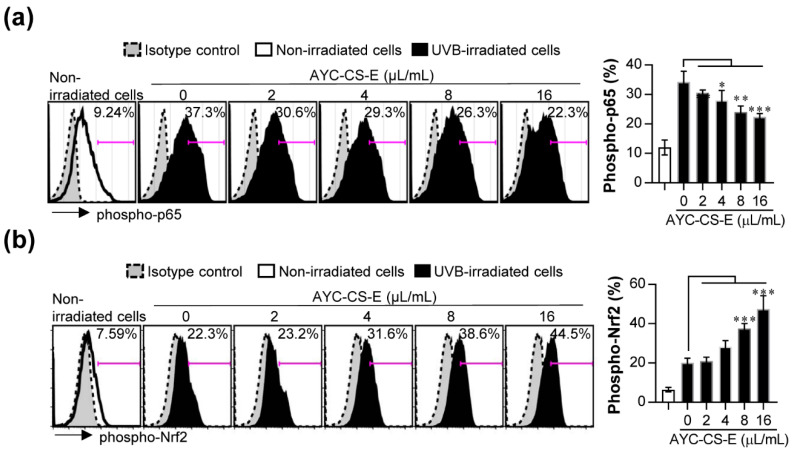
Effect of AYC-CS-E treatment on nuclear factor-kappa B (NF-κB) signal activation and nuclear factor erythroid 2-related factor 2 (Nrf2) degradation of UVB-irradiated HaCaT cells. (**a**) For phospho-NF-κB p65 analysis, HaCaT cells were treated with various concentrations (2, 4, 8, or 16 μL/mL) of AYC-CS-E (30 mg/mL) for 12 h after being exposed to 8 mJ/cm^2^ UVB irradiation, and cells were harvested. The harvested cells were fixed/permeabilized and stained with allophycocyanin (APC)-conjugated phospho-NF-κB p65 (Ser536) antibody for 30 min at 4 °C. (**b**) For phospho-Nrf2 analysis, cells were treated with various concentrations (2, 4, 8, or 16 μL/mL) of AYC-CS-E (30 mg/mL) for 3 h after being exposed to 8 mJ/cm^2^ UVB irradiation, and cells were harvested. The harvested cells were fixed/permeabilized and stained with phospho-Nrf2 (Ser40) antibody for 30 min at 4 °C, and then stained with Alexa Fluor 488-conjugated goat anti-rabbit Immunoglobulin G (IgG) antibody for 30 min at 4 °C. Protein levels of phospho-NF-κB p65 and phospho-Nrf2 were analyzed by flow cytometry. All bar graphs show the means ± standard deviation (SD) of 3 samples. One representative plot out of three independent experiments is shown. * *p* < 0.05, ** *p* < 0.01, or *** *p* < 0.001.

The nuclear factor erythroid 2-related factor 2 (Nrf2) and nuclear factor-kappa B (NF-κB) signaling pathways play key roles in cellular defense against UVB-induced oxidative damage [41,42]. The activated (phosphorylated) NF-κB p65 signals have been reported to be highly expressed in skin cells following UVB exposure and are associated with inflammatory damages [42]. In addition, Nrf2 activation (Nrf2 phosphorylation) in oxidative stress conditions contributes to the antioxidant process by regulating antioxidant gene expression and by orchestrating anti-inflammatory response [42]. Thus, the regulatory effect of phosphorylated Nrf2 and NF-κB p65 expression following AYC-CS-E treatment in UVB-irradiated HaCaT cells was investigated. Interestingly, the increase in phosphorylation of NF-κB p65 in UVB-irradiated HaCaT cells was suppressed upon treatment with AYC-CS-E (Figure 6a). Nrf2 activation (Nrf2 phosphorylation) was increased in UVB-irradiated HaCaT cells treated with AYC-CS-E compared to UVB-irradiated HaCaT cells not treated with AYC-CS-E (Figure 6b). Next, to investigate whether AYC-CS-E directly modulates the antioxidant activity by regulating the levels of the free radicals in UVB-irradiated HaCaT cells, the ability of AYC-CS-E to scavenge 2,2-diphenyl-1-picrylhydrazyl (DPPH) free radicals in cell-free condition was evaluated. As a result, AYC-CS-E did not show any DPPH free radical scavenging ability in cell-free conditions (Supplementary Figure S1), indicating that it does not have a direct inhibitory effect against free radicals such as ROS. These results suggest that AYC-CS-E treatment may contribute to the inhibition of pro-inflammatory cytokines and oxidative stress via NF-κB (inhibition of phosphorylated NF-κB p65) and Nrf2 (induction of phosphorylated Nrf2) signals in UVB-irradiated HaCaT cells, which explains the therapeutic benefit of AYC-CS-E for the anti-photoaging effect.

### 2.6. Comparison of Anti-Photoaging Efficacy between AYC-CE-E and Extract (AYC-P-E) Isolated from A. yomena Callus Pellets on UVB-Irradiated HaCaT Cells

Finally, we investigated the anti-photoaging effects between AYC-P-E and AYC-CE-E. When UVB-irradiated HaCaT cells were treated with AYC-P-E and AYC-CE-E, the degree of improvement for cell viability observed after AYC-P-E treatment (increased cell viability by 11%, 14%, and 12% at concentrations of 4, 8, and 16 μg/mL, respectively) was less than that observed after AYC-CE-E treatment (increased cell viability by 16%, 21%, and 48% at concentrations of 4, 8, and 16 μg/mL, respectively) (Supplementary Figure S2a). In addition, UVB-irradiated HaCaT cells treated with AYC-P-E showed relatively low levels of elastase inhibition, type I procollagen synthesis, and TNF-α inhibition compared to UVB-irradiated HaCaT cells treated with AYC-CS-E (Supplementary Figure S2b,c), whereas no changes were observed in the expression of other pro-inflammatory cytokines (IL-8 and IL-1β), anti-inflammatory cytokines (TGF-β and IL-10), and intracellular ROS levels (data not shown). As shown in Figure 5 and Figure 6, an increase in TGF-β production, inhibitory effect of IL-8, IL-1β production, and intracellular ROS levels was only observed in UVB-irradiated HaCaT cells treated with AYC-CS-E. Furthermore, metabolites from AYC-P-E were measured by LC/MS (Supplementary Figure S3). AYC-P-E has 19 major metabolites, including 1 isoflavonoid (robustic acid), 1 flavonoid (delphinidin 3-arabinoside), 4 indanes (pterosin C, pterosin P, acetylpterosin C, and pterosin N), 1 cinnamic acid and derivative (3,4-Dicaffeoyl-1,5-quinolactone), 1 amino acid (L-Thyronine), 3 sphingolipids (dehydrophytoshingosine, dihydrosphingosine, and phytosphingosine), 3 fatty amides (α-Linolenic acid, linoleoyl ethanolamide, and oleamide), and 5 glycerophospholipids (LysoPC(18:2), LysoPC(16:0), LysoPC(18:1), LysoPC(17:0), and LysoPC(18:0)). Importantly, there were differences in metabolite compositions between AYC-CS-E and AYC-P-E (Supplementary Table S1). These results suggest that metabolite composition of AYC-CS-E may have better potential as an effective cosmetic ingredient for anti-photoaging than AYC-P-E.

## 3. Discussion

In this study, a system to produce *A. yomena* extract by culturing the callus based on the bioreactor that can allow rapid process scale-up was built, and the effects of this extract on UVB-induced photoaging were tested. We found that 17 major metabolites that may be associated with their pharmacological activities were characterized or tentatively identified from AYC-CS-E for the first time (Figure 1 and Table 1). Importantly, AYC-CS-E alleviated photoaging by relieving cell death (Figure 2b), rescuing elasticity (Figure 3), and inhibiting pro-inflammatory response/oxidative stress (Figure 5a,b) in UVB-irradiated keratinocyte. Furthermore, AYC-CE-E isolated from culture supernatants showed better anti-photoaging effects than AYC-P-E prepared by hot water extraction method in callus pellets (Supplementary Figure S2) and revealed the differences in metabolite compositions between AYC-CS-E and AYC-P-E (Supplementary Table S1). These results suggest that metabolite composition of AYC-CS-E may have more potential as an effective cosmetic ingredient than AYC-P-E. In addition, the isolation method of AYC-CE-E extracted without moving through various processing steps is expected to be useful in isolating effective functional materials for anti-photoaging. 

Prolonged exposure to UV rays causes skin-aging symptoms, such as skin problems, spots, freckles, skin pigmentation, and skin diseases such as skin cancer, and is directly involved in the formation of wrinkles by damaging collagen and elastin, the collagen fibers in the skin [43,44,45]. The functional characteristics of human skin are closely associated with the state of collagen, the most abundant skin structural protein [46]. Collagen protects the skin from external stimuli by imparting strength and tension to the skin, and the reduction of collagen leads to skin aging and wrinkle formation [47]. Production of collagen and elastin is essential for the recovery of skin damage caused by photoaging [48]. UV rays accelerate skin aging by destroying collagen, elastin, and hyaluronic acid, which are important for skin elasticity [49,50]. Elastin and collagen are the major factors associated with skin elasticity and wrinkles [49]. As aging progresses, the synthesis of type I procollagen in the skin decreases, and the activity of MMP-1 increases [51]. Interestingly, we found that the expression of type I procollagen, the collagen precursor, increased, while that of elastinase, an enzyme inhibiting elastin synthesis, was inhibited in UVB-irradiated HaCaT cells treated with AYC-CS-E (Figure 3), suggesting that AYC-CS-E may have the potential to help recover from skin damage, skin aging, and wrinkle formation caused by UVB irradiation.

In addition, oxidative stress, defined as a state of excess ROS production, increases in cells exposed to UV radiation [52]. This results in the secretion of inflammatory cytokines in the epidermal keratinocytes, and collagen expression is suppressed in dermal fibroblasts, while collagen is destroyed due to the increased expression of MMPs, leading to the deformation of the dermal layer [48,52,53]. MMPs can be activated even upon extremely low UVB exposure, which in turn promotes photoaging reactions, such as loss of elasticity and wrinkles [52]. In addition to MMPs, HAS-2 is a major factor in the biosynthesis of hyaluronic acid, which is associated with skin elasticity and wrinkles [54]. Filaggrin, a protein that plays the most important role in the stratum corneum [55], is a micro-fibrous aggregation protein that attaches keratin intermediate microfibers, such as glue, and is an essential factor in the differentiation of the stratum corneum, formation of a skin barrier, and water retention [56]. Moreover, UV rays can cause hyper-keratinization by increasing the expression of KRT-1 in keratinocytes present in the basal layer of the epidermis, thereby causing an increase of MMP-1 expression, production of pro-inflammatory cytokines, and aging of the epidermis [57]. Therefore, the use of materials that can effectively control intracellular ROS levels, inflammatory cytokines, MMPs, filaggrin, HAS-2, and KRT-1 as cosmetic ingredients has also been reported as an effective strategy for controlling photoaging reactions. Our results suggested that treatment of UVB-irradiated HaCaT cells with AYC-CS-E effectively improved various factors (MMP-1, Figure 3c; MMP-3, MMP-9, filaggrin, HAS-2, KRT-1, Figure 4; intracellular ROS, Figure 5a; pro-inflammatory cytokines, Figure 5b) associated with oxidative stress, inflammatory reactions, skin damage recovery, skin moisture retention, and hyper-keratinization caused by photoaging at the gene and protein levels.

The representative inhibitory cytokine, TGF-β, plays a crucial role in the control of acute inflammatory response and oxidative stress directly or by downregulating antioxidative systems and modulating immune systems [58,59]. For example, the absence of TGF-β signals in skin cells can induce collagen damage by enhancing the high expression of multiple MMPs, the creation of the inflammatory dermal microenvironment by promoting the high production of multiple pro-inflammatory cytokines, and a decrease in elastin expression by regulating various signaling molecules, including protein kinase C- δ and p38 [58,60]. Moreover, TGF-β signals in the skin are essential for the maintenance of immature or tolerogenic phenotype of langerhans cells (Dendritic cells) by regulating differentiation and maturation. In inflamed skin, these cells can efficiently induce expansion and differentiation of regulatory T cells that are engaged in suppressing various immune responses, including inhibition of CD4^+^ T cell activity [61]. In fact, CD4^+^ T cells, particularly Th1 and Th17 cells to produce pro-inflammatory cytokines, cause chronic inflammatory skin diseases (e.g., atopic dermatitis and psoriasis) by leading to a sustained and amplified inflammatory status [62]. Therefore, TGF-β can play an important role in the prevention and therapy of skin photoaging and chronic inflammatory skin diseases. Interestingly, a large amount of TGF-β in UVB-irradiated keratinocytes was produced after AYC-CS-E treatment (Figure 5c), indicating that AYC-CS-E-induced anti-photoaging effects may be due to the production of TGF-β.

In addition, important signals that are regulated in response to UVB exposure are NF-κB and Nrf2, which are prime molecular targets for inducing anti-inflammatory and antioxidant activity [63,64]. The activation of NF-κB in skin cells upon UVB exposure can promote skin cell damage, pro-inflammatory cytokine production, and MMP expression [64]. In other respects, it has been reported that the Nrf2 activation (Nrf2 phosphorylation) can initiate the transcription of a battery of cytoprotective genes (including heme oxygenase-1, catalase, and superoxide dismutase), thereby increasing the anti-photoaging activity, such as induction of TGF-β production and inhibition of NF-κB signal, oxidative stress, and pro-inflammatory cytokines [41,63]. We also found that AYC-CS-E treatment decreased NF-κB activation (Figure 6a) and promoted Nrf2 phosphorylation (Figure 6b) in UVB-irradiated keratinocytes.

In summary, AYC-CS-E contained secondary phenolic metabolites, including 1 isoflavonoid (Robustic acid) and 1 flavonoid (3,5-Di-O-methyl-8-prenylafzelechin-4beta-ol). These metabolites have been reported as interesting alternative sources for pharmaceutical and medicinal applications [65,66]. However, the anti-photoaging effects of robustic acid and 3,5-Di-O-methyl-8-prenylafzelechin-4beta-ol found in this study remain unknown. Thus, while the anti-photoaging effect of these phenolic compounds and other metabolites requires more directed study, our results predicted that metabolites included in AYC-CS-E may contribute to promoting the anti-photoaging effect by inducing the inhibition of NF-κB signals and activation of Nrf2 signals and producing the anti-inflammatory cytokine TGF-β.

## 4. Materials and Methods

### 4.1. Plant Sterilization and Callus Induction

**Figure 7:**
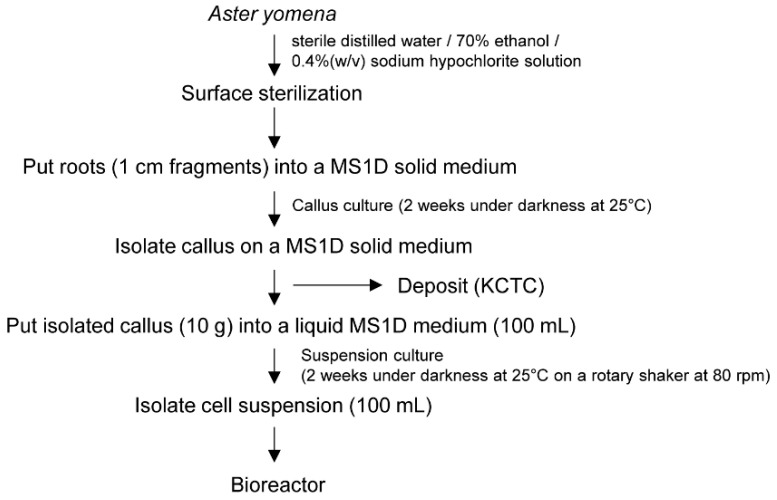
Schematic representation of the procedure for the establishment of a callus culture from *A. yomena* roots.

*A. yomena* used in this study were harvested from Jeongeup-si, Jeollabuk-do, Republic of Korea. For sterilization, the collected *A. yomena* were washed under running water for 30 min, and then the moisture was removed. Samples were surface-sterilized by soaking with 70% ethanol for 1 min and rinsed two times in sterile distilled water. In addition, 0.4% (*w/v*) sodium hypochlorite solution was added, and surface sterilization was performed for ~20 min while shaking. The sample was rinsed three times in sterile distilled water. After sterilization, the roots were cut into 1 cm long fragments and then cultured on a MS1D (Murashige and Skoog basal medium supplemented with 1 mg/L 2,4-dichlorophenoxyacetic acid (2,4-D), 3% (*w/v*) sucrose, and 0.4% (*w/v*) Gelrit) solid medium for another 2 weeks under darkness at 25 °C. The cultured callus made in this way was deposited with Korean Collection for Type Cultures (KCTC; http://bioproduct.kribb.re.kr, accessed on 10 March 2021) as bio-product KCTC PC4338. For suspension culture, 10 g of callus cultured for 2 weeks was inoculated into a 500 mL flask containing 100 mL of a liquid MS1D medium. The pH of the medium was adjusted to 5.8 using 1 N KOH. Cell suspensions were cultured in darkness at 25 °C on a rotary shaker at 80 rpm, and the callus was used for bioreactor culture (Figure 7).

### 4.2. Bioreactor Experiment 

The exponential phase 100 mL cell suspension contained in each of the three flasks was transferred to a 2 L tank of a sterilized glass bioreactor, and the volume was adjusted to 1.5 L with the MS1D medium. The bioreactor was maintained at 25 °C in darkness, and the callus was incubated for 10 days. After incubation, culture supernatants and pellets were divided via centrifugation for 30 min at 12,000 rpm. For AYC-CS-E extraction, the collected solutions were dried in a vacuum freeze drier (VD-800F; Taitec, Saitama-ken, Japan), and freeze-dried powders were resuspended in phosphate-buffered saline (PBS, Biowest, Nuaille, France) and filtered through a 0.22 μm filter (Corning, Steuben county, NY, USA). The final concentration of AYC-CS-E was 30 mg/mL (yield, 11%). For AYC-P-E extraction, collected pellets (5 g) were suspended in 1 L sterile distilled water and incubated at 80 °C for 2 h. Collected solutions were dried in a vacuum freeze drier, and freeze-dried powders were resuspended in PBS and filtered through a 0.22 μm filter. The final concentration of AYC-P-E was 30 mg/mL (yield, 56.1%). AYC-CS-E and AYC-P-E were stored at −80 °C until use.

### 4.3. Ultra-Performance Liquid Chromatography-Quadrupole Time-of-Flight/Mass Spectrometry (UPLC-QTOF/MS) Analysis

The samples were injected in an Acquity UPLC BEH C18 column (2.1 × 100 mm, particle size 1.7 μm; Waters, Milford, MA, USA) equilibrated with water containing 0.1% formic acid and eluted in a gradient with acetonitrile (ACN) containing 0.1% formic acid at a flow rate of 0.35 mL/min for 9 min. The eluted metabolites were analyzed by QTOF/MS in electrospray ionization (ESI)-positive mode. The capillary voltages and sampling cones were set at 3 kV and 30 V, respectively. The source temperature was set to 100 °C, the desolvation flow rate was 800 L/h, and the desolvation temperature was 400 °C. The TOF MS data were collected in the m/z 50–1500 range with a scan time of 0.2 s. The MS/MS spectra of the extracts were collected in the 50–1000 range by a collision energy ramp from 10 to 30 eV. Leucine-enkephalin (*m/z* 556.2771 for positive mode) was used at a frequency of 10 s. Acquired MS data included m/z, retention time, and ion intensity, and all data were extracted with UNIFI software (Waters). Collection, normalization, and alignment of the MS datasets analyzed by UPLC-QTOF/MS were obtained using UNIFI software (Waters, Milford, MA, USA). The metabolites were identified using the Human Metabolome Database (HMDB) (www.hmdb.ca, accessed on 10 March 2021), Metabolite and Chemical Entity (METLIN) database (metlin.scripps.edu, accessed on 10 March 2021), chemspider (www.chemspider.com, accessed on 10 March 2021), literature references, and authentic standards.

### 4.4. Cell Culture

The HaCaT cell line was provided by the Korean Cell Line Bank (Seoul, Korea) and cultured in Dulbecco’s modified Eagle’s medium (DMEM, Gibco BRL, Grand Island, NY, USA) supplemented with 10% fetal bovine serum (Gibco BRL) and antibiotics (100 U/mL streptomycin and 100 U/mL penicillin, Gibco BRL) in an incubator at 37 °C and 5% CO_2_. Passage 3 (P3) cells were used for all experiments.

### 4.5. Cell Viability

To examine the cell viability after treatment with AYC-CS-E alone, HaCaT cells at a concentration of 1 × 10^5^ cells/well were seeded in a 48-well plate, incubated for 12 h in an incubator at 37 °C and 5% CO_2_, and treated with various volumes (2 to 32 μL/mL) of AYC-CS-E alone. In addition, to investigate the effect of AYC-CS-E on the viability of UVB-irradiated HaCaT cells, DMEM was removed, and cells were washed twice with PBS. After adding 1 mL PBS to the cell culture dish to prevent drying during UVB irradiation, the cells were irradiated with 8 mJ/cm^2^ UVB, which would affect cell proliferation. After UVB irradiation, the cells were washed once with 1 mL PBS, and a culture solution containing AYC-CS-E or AYC-P-E (0.125 to 64 µL/mL) was added. UV irradiation of cells was performed using a UVB lamp of 306 nm (Dongseo Science, Korea), and the amount of UV light was measured using a UVA/UVB light meter 850009 (Sper Scientific, Scottsdale, AZ, USA). Cell viability was confirmed 24 h after treatment with AYC-CS-E or AYC-P-E. Cell viability was measured by adding 20 μL of 3-(4, 5-dimethylthiazol-2-yl)-2, 5-diphenyl-tetrazolium bromide (MTT; Sigma-Aldrich, St. Louis, Mo, USA) reagent prepared at a concentration of 5 mg/mL to each sample, re-culturing for 30 min, removing the culture solution, and adding 200 μL dimethyl sulfoxide (DMSO) to each well to let the reaction occur at room temperature for 10 min. Absorbance was measured at 540 nm using a microplate reader (Molecular Devices Inc., San Jose, CA, USA).

### 4.6. Measurement of Elastase Activity

Elastase activity was measured using N-succinyl-tri-alanyl-p-nitroanilide (N-STANA, elastase substrate), as previously reported, with several modifications [67]. Each cell group (non-irradiated HaCaT cells, UVB-irradiated HaCaT cells, UVB-irradiated HaCaT cells treated with AYC-CS-E, and UVB-irradiated HaCaT cells treated with AYC-P-E) was collected and dissolved in 0.1% Triton X-110 in 0.2 M Tris-HCl buffer (pH 8.0). Next, the cells were repeatedly frozen and thawed three times, and the cell debris was removed via microcentrifugation (3000 rpm, 20 min, 4 °C), and the remaining samples containing proteins were used. After adding 100 μg of the protein sample into the 96-well plate for each group, the volume of each well was adjusted to 98 μL with 0.2M Tris-HCl. Two microliters of 50 mM N-STANA (elastase substrate; Peptide Institute Inc, Osaka, Japan) solution was dispensed into each well, and then the mixture was incubated at 37 °C for 90 min. Elastase activity was determined by measuring the absorbance at 405 nm using a microplate reader.

### 4.7. Quantitative Real-Time Polymerase Chain Reaction (qRT-PCR)

The cells were irradiated with 8 mJ/cm^2^ UVB and then washed once with 1 mL of PBS. A culture solution containing AYC-CS-E (0.125 to 64 µL/mL) was added next. After 24 h, the cultured cells were collected, and total ribonucleic acid (RNA) was extracted using the TRIzol reagent (Life Technologies; Carlsbad, CA, USA) according to the manufacturer’s instructions. Only RNA with a 260/280 nm ratio of 1.8 or higher, determined with Nanodrop (Maestrogen, Ramsey, MN, USA), was used. Complementary DNA (cDNA) was synthesized using 1 μg RNA and a qPCRBio cDNA Synthesis Kit (PCR Biosystems, Staffordshire, UK). Next, to measure the expression of genes, SYBR Green Mastermix (Faststart Universial SYBR Green Mastermix, Roche, Nutley, NJ, USA) and the respective primers were used to perform qRT-PCR on a real-time PCR machine (Eco 48 real-time PCR system; PCRmax, Staffordshire, UK), and the PCR was run for 40 cycles. Table 2 shows the sequences of PCR primers for each gene. A total of 20 μL of the real-time PCR reaction solution was used, consisting of 5 μL cDNA, 10 μL qPCRBIO SyGreen Blue Mix Lo-ROX (PCR Biosystems), 0.5 μL forward reverse primer, and 4 μL distilled water. The PCR steps for all genes were as follows: For a hot start, 40 cycles of 95 °C for 5 min, 95 °C for 20 s, 60 °C for 20 s, and 72 °C for 30 s, melting curve at 95 °C for 1 min, 55 °C for 1 min, and 30 °C for 1 min. The expression of each gene was normalized to that of β-actin, as a housekeeping gene.

### 4.8. Measurement of Intracellular ROS Level

The cells were irradiated with 8 mJ/cm^2^ UVB, and then the cells were washed once with 1 mL PBS, and a culture solution containing AYC-CS-E (0.125 to 64 µL/mL) was added. After 24 h, cells were harvested and stained with 5 μM 2′,7′-dichlorofluorescein diacetate (DCFH-DA; Sigma-Aldrich) at 37 °C for 30 min and washed twice with PBS. The intracellular ROS expression levels (DCFH-DA-positive cells) of each cell were analyzed with a Life Launch Attune Nxt Flow Cytometer (ThermoFisher Scientific, Waltham, MA, USA) using FlowJo software (Version 10; Tree Star, Inc., Ashland, OR, USA).

### 4.9. Measurement of Intracellular Nrf2 and NF-κB Signals

The cells were irradiated with 8 mJ/cm^2^ UVB and then were washed once with 1 mL of PBS. Next, a culture solution containing AYC-CS-E (0.125 to 64 µL/mL) was added. After 3 h (for phospho-Nrf2 analysis) or 12 h (for phospho-NF-κB p65 analysis), cells were fixed and permeabilized with a Cytofix/Cytoperm kit (BD Bioscience, San Diego, CA, USA) for 30 min at 4 °C and washed twice with BD Perm/Wash buffer. For Nrf2 phosphorylation analysis, each cell was stained with phospho-Nrf2 (Ser40) monoclonal antibody (ThermoFisher Scientific) for 30 min at 4 °C and then stained with Alexa Fluor 488-conjugated goat anti-rabbit IgG antibody for 30 min at 4 °C. For NF-κB p65 analysis, each cell was stained with APC-conjugated phospho-NF-κB p65 (Ser536) antibody (ThermoFisher Scientific) for 30 min at 4 °C. The intracellular phospho-Nrf2 and phospho-NF-κB expression levels of each cell were analyzed with a Life Launch Attune Nxt Flow Cytometer using FlowJo software.

### 4.10. Enzyme-Linked Immunosorbent Assay (ELISA)

The cells were irradiated with 8 mJ/cm^2^ UVB and then were washed once with 1 mL of PBS. Next, a culture solution containing AYC-CS-E or AYC-P-E (0.125 to 64 µL/mL) was added. After 24 h, type I procollagen, matrix metalloproteinase-1 (MMP-1), tumor necrosis factor alpha (TNF-α), IL-8, and IL-1β levels were measured in the culture medium. Type I procollagen and MMP-1 levels were measured using the Human Pro-Collagen I alpha 1 ELISA Kit and Fluorokine E Human Active MMP-1 ELISA Kit according to the manufacturer’s instructions (Abcam, Cambridge, UK). In addition, the levels of TNF-α, IL-8, IL-β, TGF-β, and IL-10 were measured at 450 nm using a microplate reader according to the manufacturer’s instructions, using an ELISA kit from R&D Systems.

### 4.11. DPPH Radical Scavenging Assay

The stable DPPH free radical was purchased from Sigma-Aldrich. To determine the DPPH radical scavenging activity, 100 μL of AYC-CS-E (2, 4, 8, or 16 μL/mL) and Vitamin C (1 mM, used as a positive control for antioxidant activity) solutions was added to 100 μL of DPPH solution (0.1 mM, dissolved in methanol). After incubating the mixture at room temperature in the dark for 15 min, the absorbance was measured at 517 nm using a microplate reader.

### 4.12. Statistics

The levels of significance for comparison between samples were determined using one-way analysis of variance (ANOVA) followed by Tukey’s multiple comparison test using GraphPad statistical software (GraphPad Software, San Diego, CA, USA). 

## 5. Conclusions

While previous studies have demonstrated the beneficial anti-allergic effects of *A. yomena* extracts, specific process steps were required, including hot water extraction and ethanol precipitation, for commercial use as functional food [23]. Importantly, these steps can cause denaturation and loss of functional materials. Therefore, because our system does not require these process steps, the *A. yomena* callus culture system established in this study is expected to be useful in providing effective functional materials for anti-photoaging. This prediction can be seen from the results that AYC-CS-E can induce a better anti-photoaging activity than AYC-P-E.

In addition, further, more detailed studies on the characteristics of *A. yomena* callus culture medium need to be conducted by isolating the extracellular vesicles and identifying the most effective cosmetic ingredients. In fact, extracellular vesicles can be secreted into the medium during callus growth and contain a variety of physiologically active substances [68]. Thus, we predict that AYC-CS-E-mediated anti-photoaging effects can be induced by extracellular vesicles contained in the callus culture medium. In conclusion, our research results can be important data for the development of effective cosmetic ingredients that can help in the treatment of various skin diseases associated with photoaging caused by UVB exposure.

## Figures and Tables

**Table 1:** Metabolites of extract (AYC-CS-E) isolated from culture supernatant of *Aster yomena* callus analyzed by electrospray (ESI)-positive mode in ultra-performance liquid chromatography-quadrupole-time-of-flight mass spectrometry (UPLC-QTOF/MS).

Peak No.	Retention Time (min)	Identification	Exact Mass (*m/z*)	Fragment Ions (*m/z*)
1	0.60	Robustic acid	381.07	349, 251, 233, 175
2	1.92	3,4-Dicaffeoyl-1,5-quinolactone	499.12	319, 163
3	3.18	Acetylpterosin C	277.10	235, 217, 131
4	4.42	Pterosin N	235.09	217, 175, 147, 91
5	5.28	L-Thyronine	274.27	256, 230
6	5.74	3,5-Di-O-methyl-8-prenylafzelechin-4beta-ol	387.18	147, 105
7	5.98	Dehydrophytosphingosine	316.28	298, 280
8	6.28	Phytosphingosine	318.30	300, 270, 155
9	7.69	LysoPC(18:2)	520.33	502, 337, 184, 104
10	7.78	α-Linolenic acid	279.23	261, 243, 109, 95, 81
11	7.93	LysoPC(16:0)	496.33	478, 313, 184
12	8.17			478, 313, 184, 104
13	8.45	LysoPC(18:1)	522.35	504, 184, 104
14	9.40	LysoPC(18:0)	524.36	506, 341, 184, 104
15	10.03	Palmitic amide	256.26	186
16	10.23	Oleamide	282.27	265, 247
17	11.88	13Z-Docosenamide	338.34	321, 303
18	14.01	PC(18:2/16:0)	758.57	575, 337, 184

**Table 2:** Primers used in this study.

Primers	Forward Primer Sequence (5‘ to 3’)	Reverse Primer Sequence (5‘ to 3’)
Filaggrin	ATCTTCTCGGGAGCAGTCAA	ACCCGGATTCACCATAATCA
HAS-2	CCTGGGCTATGCAACAAAAT	TAAGGCAGCTGGCAAAAGAT
KRT-1	AAGCTGAATGACCTGGAGGA	ACCTCCACTGATGGTGGTGT
MMP-3	CTTTCCTFFCATCCCGAAGT	GCATAGGCATGGGCCAAAAC
MMP-9	GTGCTCCTGGTGCTGGGCTG	GGTGCCACTTGAGGTCGCCC
β-actin	GCACCACACCTTCTACAATG	TGCTTGCTGATCCACATCTG

## Data Availability

Data available in a publicly accessible repository.

## Supplementary Materials

The following are available online at https://www.mdpi.com/article/10.3390/plants10040659/s1, Figure S1: Antioxidant activity of AYC-CS-E in cell-free condition, Figure S2: Effect of AYC-CS-E and AYC-P-E treatment on cell viability, elastase inhibition, type I procollagen expression of UVB-irradiated HaCaT cells, Figure S3: Representative ultra-performance liquid chromatography-quadrupole-time-of-flight mass spectrometry (UPLC-QTOF/MS) extract (AYC-P-E) isolated from *Aster yomena* callus pellets, Table S1: Identification of metabolites from AYC-CS-E and AYC-P-E analyzed by ESI-positive mode in UPLC-QTOF/MS.

## Abbreviations

AYC-CS-E	Extract isolated from culture supernatant of *Aster yomena* callus
AYC-P-E	Extract isolated from *Aster yomena* callus pellet
UVB	Ultraviolet B
qRT-PCR	Quantitative real-time polymerase chain reaction
UPLC	Ultra-performance liquid chromatography
Q-TOF-MS	Quadrupole time-of-flight mass spectrometry
MMP-1	Matrix metalloproteinase-1
MMP-3	Matrix metalloproteinase-3
MMP-9	Matrix metalloproteinase-9
HAS-2	Hyaluronic acid synthase 2
KRT-1	Keratin 1
LC/MS	Liquid chromatography/mass spectrometry
ROS	Reactive oxygen species
DCFH-DA	2′,7′-dichlorofluorescein diacetate
ELISA	Enzyme-linked immunosorbent assay
TNF-α	Tumor necrosis factor-α
IL-8	Interleukin-8
IL-1β	Interleukin-1 beta
TGF-β	Transforming growth factor-beta
IL-10	Interleukin-10
ACN	Acetonitrile
DMEM	Dulbecco’s modified Eagle’s medium
DMSO	Dimethyl sulfoxide
N-STANA	N-succinyl-tri-alanyl-p-nitroanilide
RNA	Ribonucleic acid
cDNA	Complementary DNA
DPPH	2,2-diphenyl-1-picrylhydrazyl
