# Supplementary material for: The Metabolite Profile in Culture Supernatant of Aster yomena Callus and Its Anti-Photoaging Effect in Skin Cells Exposed to UVB
Source: Plants (Basel). 2021 Mar 30;10(4):659. doi: 10.3390/plants10040659 (PMC8066191; doi:10.3390/plants10040659)
Supplement: Supplementary file 1 [file plants-10-00659-s001.pdf]

plants

Article

The metabolite profile in culture supernatant of *Aster yomena* callus and its anti-photoaging effect in skin cells exposed to UVB

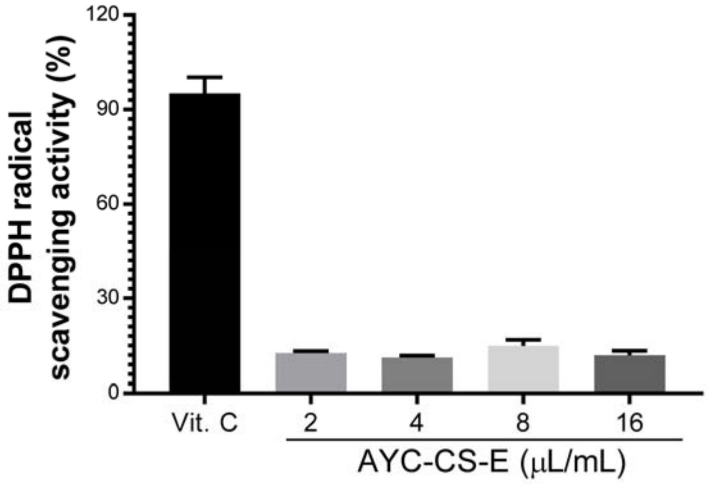

Supplementary Figure S1: Antioxidant activity of AYC-CS-E in cell-free condition. The antioxidant activity of AYC-CS-E and Vitamin C (Vit. C, 1 mM). Analysis of DPPH radical was performed as described in the *Materials and Methods* section. All bar graphs show the means ± standard deviation (SD) of 3 samples. One representative plot out of three independent experiments is shown.

Supplementary Figure S2: Effect of AYC-CS-E and AYC-P-E treatment on cell viability, elastase inhibition, type I procollagen expression of UVB-irradiated HaCaT cells. (**a-c**) HaCaT cells were treated with various concentrations (2, 4, 8, or 16 μL/mL) of AYC-CS-E (30 mg/mL) and AYC-P-E (30 mg/mL) for 24 h after being exposed to 8 mJ/cm² UVB irradiation. (**a**) The cell viability for each conditions was measured using MTT assay. (**b**; raft panel) Elastase inhibition levels were measured in each conditions via elastase substrate (N-STANA) treatment, as described in the *Materials and Methods* section. Type I procollagen levels (**b**; right panel) and TNF-α levels (**c**) in the culture supernatants were analyzed using each enzyme-linked immunosorbent assay (ELISA) kits. All bar graphs show the means ± standard deviation (SD) of 3 samples. One representative plot out of three independent experiments is shown; *$p < 0.05$, **$p < 0.01$, or ***$p < 0.001$.

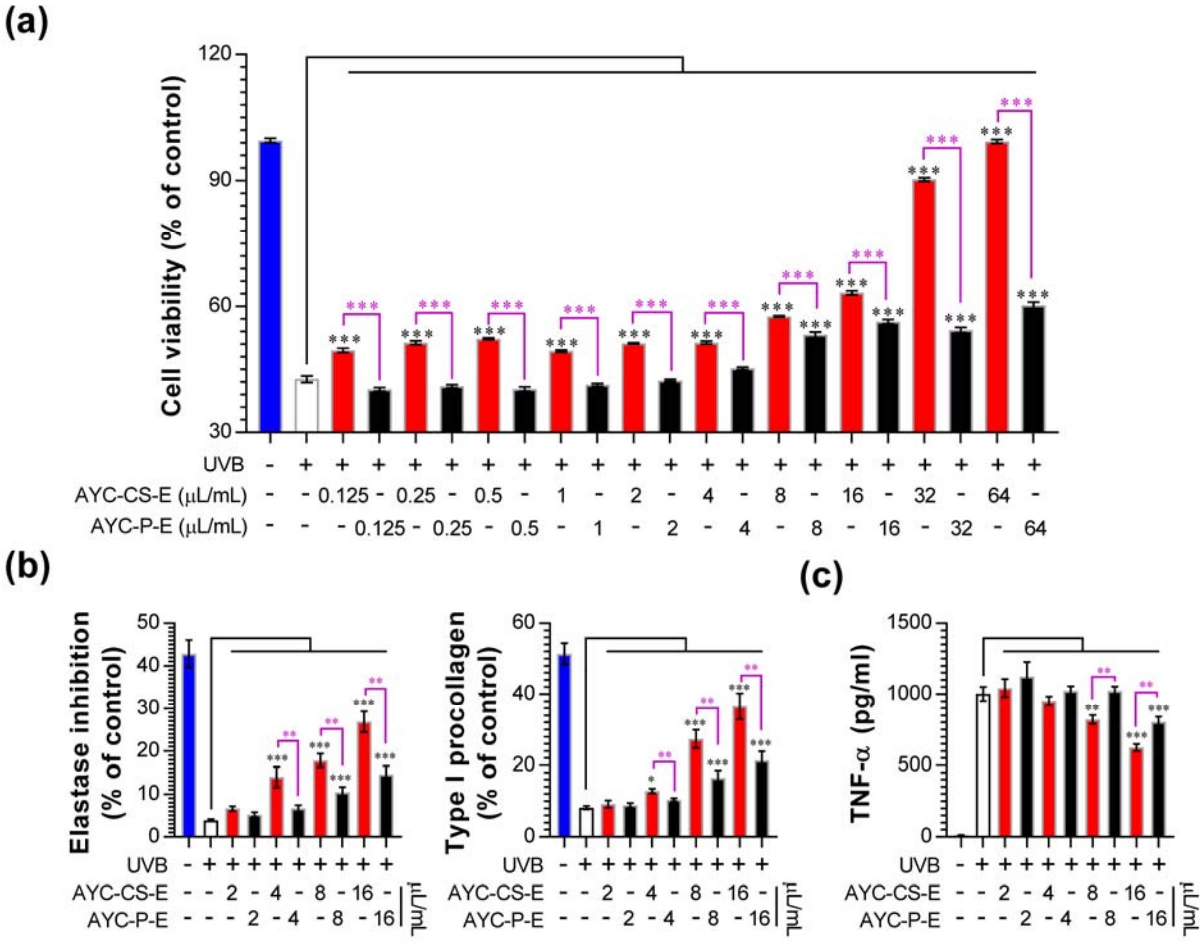

Supplementary Figure S3: Representative ultra-performance liquid chromatography-quadrupole-time-of-flight mass spectrometry (UPLC-QTOF/MS) extract (AYC-P-E) isolated from *Aster yomena* callus pellets. For analysis, the metabolites were analyzed with a BEH C18 column (2.1 x 100 mm, 1.7 μm). The eluted metabolites were analyzed by Q-TOF MS in ESI-positive mode. The UPLC-QTOF/MS chromatogram shows the following: 1, Robustic acid; 2, Delphinidin 3-arabinoside; 3, Pterosin C; 4, 3,4-Dicaffeoyl-1,5-quinolactone; 5, Pterosin P; 6, Acetylpterosin C; 7, Pterosin N; 8, L-Thyronine; 9, Dehydrophytosphingosine; 10, Dihydrosphingosine; 11, Phytosphingosine; 12, LysoPC(18:2); 13, α-Linolenic acid; 14 and 15, LysoPC(16:0); 16, LysoPC(18:1); 17, LysoPC(17:0); 18, Linoleoyl ethanolamide; 19; LysoPC(18:0); and 20, Oleamide.

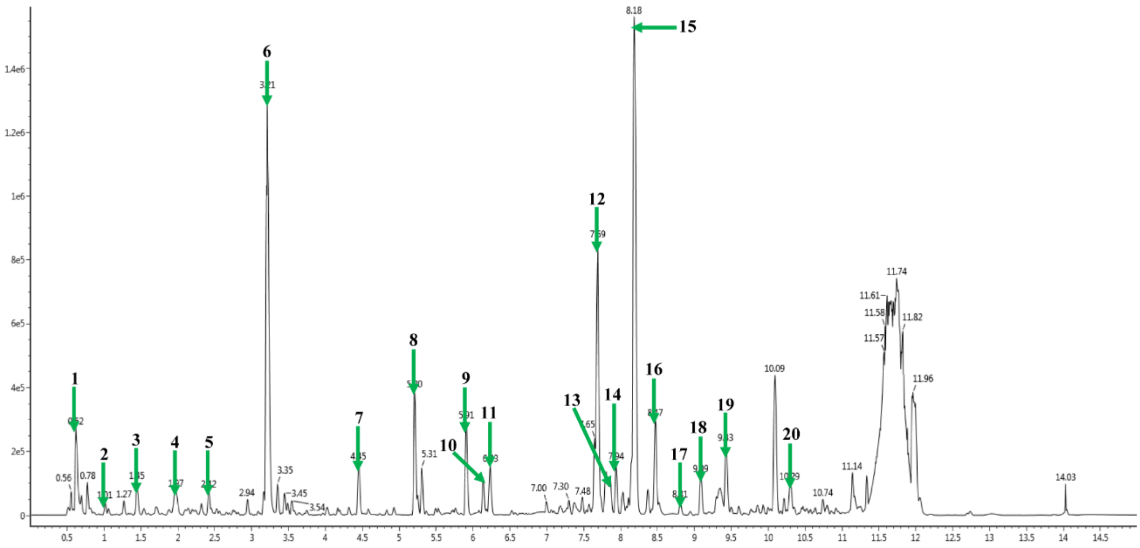

Supplementary Table S1: Identification of metabolites from AYC-CS-E and AYC-P-E analyzed by ESI-positive mode in UPLC-QTOF/MS

No.	RT (min)	Identification	Exact mass (m/z)	Fragment ions (m/z)	AYC-P-E	AYC-CS-E
1	0.60 / 0.62	Robustic acid	381.07	349, 251, 233, 175	O	O
2	1.01	Delphinidin 3-arabinoside	435.13	419, 115, 91	O	X
3	1.45	Pterosin C	235.10	217, 175, 147, 131	O	X
4	1.92 / 1.97	3,4-Dicaffeoyl-1,5-quinolactone	499.12	319, 163	O	O
5	2.42	Pterosin P	235.10	217, 191, 175, 147	O	X
6	3.18 / 3.21	Acetylpterosin C	277.12	235, 217, 175, 131	O	O
7	4.42 / 4.45	Pterosin N	235.09	217, 175, 147, 91	O	O
8	5.20 / 5.28	L-Thyronine	274.27	256, 230	O	O
9	5.74	3,5-Di-O-methyl-8-prenylafzelechin-4beta-ol	387.18	147, 105	X	O
10	5.91 / 5.98	Dehydrophytosphingosine	316.28	298, 280	O	O
11	6.14	Dihydrosphingosine	302.32	284	O	X
12	6.23 / 6.28	Phytosphingosine	318.30	300, 282, 155	O	O
13	7.69	LysoPC(18:2)	520.36	502, 337, 184, 104	O	O
14	7.78 / 7.83	α-Linolenic acid	279.24	261, 243, 109, 95, 81	O	O
15	7.93/ 7.94 / 8.17 / 8.18	LysoPC(16:0)	496.33	478, 313, 184, 104	O	O
16	8.45 / 8.47	LysoPC(18:1)	522.38	504, 184, 104	O	O
17	8.81	LysoPC(17:0)	510.38	492, 184, 104	O	X
18	9.09	Linoleoyl ethanolamide	324.30	306, 263	O	X
19	9.40 / 9.43	LysoPC(18:0)	524.36	506, 341, 184, 104	O	O
20	10.03	Palmitic amide	256.26	186	X	O
21	10.23 / 10.29	Oleamide	282.27	265, 247	O	O
22	11.88	13Z-Docosenamide	338.34	321, 303	X	O
23	14.01	PC(18:2/16:0)	758.57	575, 337, 184	X	O
